# Protective Role of *Tinospora cordifolia* against Lead-induced Hepatotoxicity

**DOI:** 10.4103/0971-6580.68343

**Published:** 2010

**Authors:** V. Sharma, D. Pandey

**Affiliations:** Bioscience and Biotechnology Department, Banasthali University, Banasthali - 304 022, Tonk, Rajasthan, India

**Keywords:** Biochemical changes, histopathology, lead nitrate, liver, mice, *Tinospora cordifolia*

## Abstract

The importance of *Tinospora cordifolia* stem and leaves extract was investigated for its possible hepatoprotective effect in Swiss albino male mice against lead nitrate induced toxicity. Oral administration of plant extracts prevented the occurrence of lead nitrate induced liver damage. The decreased level of tissue enzymes, i.e., superoxide dismutase (SOD), catalase (CAT) and increased level of aspartate aminotransferase (AST), alanine aminotransferase (ALT), alkaline phosphatase (ALP), and acid phosphatase (ACP) were observed in mice treated with lead. Administration of aqueous stem extract (400 mg/kg body weight, orally) and aqueous leaves extract (400 mg/kg body weight, orally) along with the lead nitrate (5 mg/kg body weight, i.p. for 30 days) increased the activities of SOD and CAT and decreased the levels of AST, ALT, ALP, and ACP enzymes in mice. These biochemical observations were supplemented by histopathology/histological examinations of liver section. Results of this study revealed that plant extract could afford protection against lead-induced hepatic damage.

## INTRODUCTION

Guduchi [*Tinospora cordifolia* (Willd) Miers] is a large, glabrous, deciduous climbing shrub belonging to the family Menispermacea.[1,2] It is distributed throughout tropical India subcontinent and China, ascending to an altitude of 300 m. In Hindi it is commonly known as Giloy. The whole plant is used for therapeutic purposes. It is widely used in veterinary folk medicine/ayurvedic system of medicine for its general tonic, antiperiodic, antispasmodic, anti-inflammatory, antistress, antiarthritic, antiallergic, antidiabetic, antioxidant, antileprotic, antimalarial, hepatoprotective, immunomodulatory, and antineoplastic activities.[2–7]

Despite the fact that Guduchi has myriad of medicinal properties, no attempt was made to study its regulatory role in the lead toxicity, if any. Lead has been identified as a ubiquitous environmental pollutant.[8–10] It is also known to produce reactive oxygen species resulting in lipid peroxidation, DNA damage, depletion of –SH groups.[11–13] Many organ systems largely account for its toxic action, liver is one of them.[14] Until now, the studies regarding regulation of lead toxicity are restricted to some chelating agents[12,15] and few antioxidants such as vitamin C and E.[16,17] Present investigation attempts to reveal the efficacy of the stem and leaves extract of *T. cordifolia*, if any in regulation of lead nitrate induced biochemical and histopathological changes in the liver of male albino mice.

## MATERIALS AND METHODS

### Chemicals

Lead nitrate Pb(NO_3_)_2_ and all other chemicals used in this study were of analytical grade and were purchased from reliable firms like SRL and BDH chemical (India), MERCK (Germany).

### Preparation of crude extract of *T. cordifolia*

The plant was collected from the medicinal garden of Banasthali University, Rajasthan, India. It was identified as *T. cordifolia* by a plant taxonomist. The plant materials (stem and leaves) were thoroughly washed with distilled water, shade dried and cut into small pieces, and powdered separately using laboratory homogenizer. Known quantities of these powdered materials were extracted separately using distilled water as a solvent. The extracts were then filtered through filter paper and concentrated on water bath. Finally after complete evaporation of the solvent, the residues were weighed and stored at 4°C and used to treat the animals as needed.

### Maintenance of animals

Male Swiss albino mice weighing approximately 15–30 g were obtained from Haryana agricultural university, Hissar, India. The animals were acclimatized for 7 days prior to experiment. The institutional ethics committee approved the experimental protocols. All the animals used in this study were placed in stainless steel cages in an air conditioned room maintained at temperature of 25°C±30°C and 12 h light and dark schedule. Throughout the experiment, the animals were provided standard food pellet (Hindustan lever ltd.) and water *ad libitum*. Essential cleanliness conditions were also maintained.

### Experimental protocol

Mice were divided into six groups of six each and the groups were as follows

- Group I – Control animals (no treatment)
- Group II – Lead nitrate (5 mg/kg body weight)
- Group III – TC stem extract (400 mg/kg body weight)
- Group IV – TC leaves extract (400 mg/kg body weight)
- Group V – TC stem extract (400 mg/kg body weight) + lead nitrate (5 mg/kg body weight)
- Group VI – TC leaves extract (400 mg/kg body weight) + lead nitrate (5 mg/kg body weight)

All the above groups except group I were treated once daily for the period of 30 days. After the administration of the last dose, the animals were given rest overnight and then on the next day, they were sacrificed under light ether anesthesia. The liver was removed, cleaned, and washed with phosphate buffer saline (pH 7.4) for various biochemical variables, metal analysis, and histopathology/histological studies. A small portion of liver tissue from each mouse was fixed in Bouines solution for histopathology/histological studies and remaining part was stored at –20°C for biochemical analysis and for lead nitrate estimation.

### Biochemical assay

The tissue SOD activity was assayed according to the method of Marklund and Marklund[18] and catalase activity (CAT) was determined by decomposition of H_2_O_2_ by Aebi’s method.[19] Tissue enzymes aspartate aminotransferase (AST) and alanine aminotransferase (ALT) were assayed by the method of Reitman and Frankel,[20] and alkaline phosphatase (ALP) and acid phosphatase (ACP) were determined using the method of King and Armstrong.[21]

### Histopathology/histological analysis

Histological evaluation of liver was done according to the method of Mc Manus and Mowry.[22] Liver fragments removed from the mice were fixed in Bouines solution, dehydrated in an ethanol series, and embedded in paraffin wax for histological procedure. Liver was cut in order to obtain representative section of all liver lobules. Paraffin sections (6 *μ*m thick) were stained with hematoxylen and eosin. Liver histological changes were studied from the stained sections at microscope level.

### Lead estimation

Lead concentration in liver was measured after wet acid digestion using a microwave digestion system (model MDS-2100, CEM, Matthews, CT). Lead was estimated using a hydride vapor generation system (model MHS-10, Perkin Elmer) fitted with an atomic absorption spectrophotometer (model A Analyst 100, Perkin Elmer).

### Statistical analysis

Data are expressed as the mean ± SE. Statistical analysis was done using analysis of variance (ANOVA). The level of significance was set at *p* < 0.05.

## RESULTS

The animals in all groups did not show any abnormal behavior except that lead-exposed animals were less active and more irritable. Also no significant differences were registered in the body weights of the animals from different group treatments, and none of them died during experimental period.

### Biochemical assays

Lead exposure produced significant decrease (*P*<0.05) in SOD (2.27±0.16 units/min/mg protein) and CAT (117±2.22 *μ*m of H_2_O_2_ consumed/min/mg protein) and significant increase (*P*<0.05) in AST (5.67±0.19 per min per mg protein), ALT (7.80±0.15 per min per mg protein), ALP (18.1±0.54 one king Armstrong unit/1 UI-1), and ACP (5.6±0.07 one king Armstrong unit/1 UI-1). Lead exposure also caused significant accumulation (*P*<0.05) of lead in liver tissue of mice.

*T. cordifolia* stem extract showed significant effect (*P*<0.05) on CAT and moderate (insignificant) effect on SOD. TC leaf extract showed no effect on CAT and SOD while both extracts showed moderate (insignificant) effect on AST, ALT, ALP, and ACP as compared to untreated animals.

Administration of both extracts of TC individually showed no effect on liver lead concentration.

Coadministration of TC stem and leaf extract along with lead significantly increased (*P*<0.05) the CAT (161±4.99; 153±1.34 *μ*m of H_2_O_2_ consumed/min/mg protein) while significant (*P*<0.05) fall in AST (3.11±0.49; 3.21±0.08 per min per mg protein), ALT (4.67±0.05; 4.04±0.22 per min per mg protein), ALP (13.2±0.33; 11.7±0.26 one king Armstrong unit/1 UI-1), and ACP (2.46±0.01; 2.23±0.06 one king Armstrong unit/1 UI-1) were observed with both extracts as compared to lead-treated group. Significant increase (*P*<0.05) in SOD (4.83±0.06 units/min/mg protein) with TC stem + lead was also observed but some effect on SOD was also noticed with the TC leaf + lead combination. These changes were accompanied by a significant depletion (*P*<0.05) of hepatic lead concentration in animals concomitantly administered lead and TC extracts.

### Histopathological/histological studies

Histological evaluations of liver tissue in different groups were done in the sections stained with hematoxylen and eosin.

**Figure 1:**
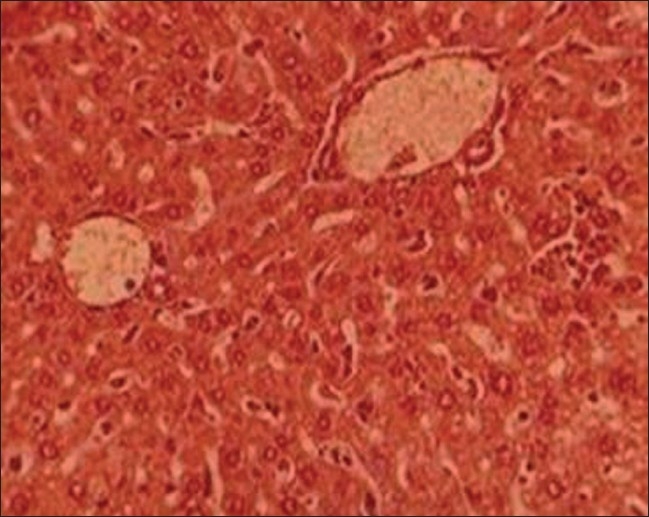
T.S. liver of control group I (H and E ×66)

*Group I (control, untreated, normal animals)*: Liver of these animals displayed normal architecture, where typical aspects, i.e., lobular pattern with prominent central vein and patient sinusoids were evident [Figure 1].

**Figure 2:**
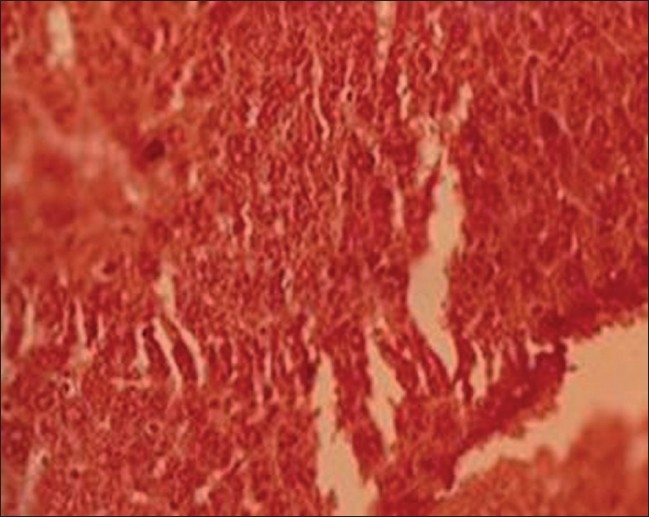
T.S. liver of lead-treated group II (H and E ×66)

*Group II (lead-treated animals)*: Lead exposure produced pronounced hepatic histopathological alterations in liver including focal necrosis with inflammatory cells, congestion at places, sinusoids not patent, centrilobular swelling, hepatocyte vacuolation and swelling, parenchyma disorganization, dilation of the inter hepatocyte space, and hemorrhagic clots when compared with group I [Figure 2].

**Figure 3:**
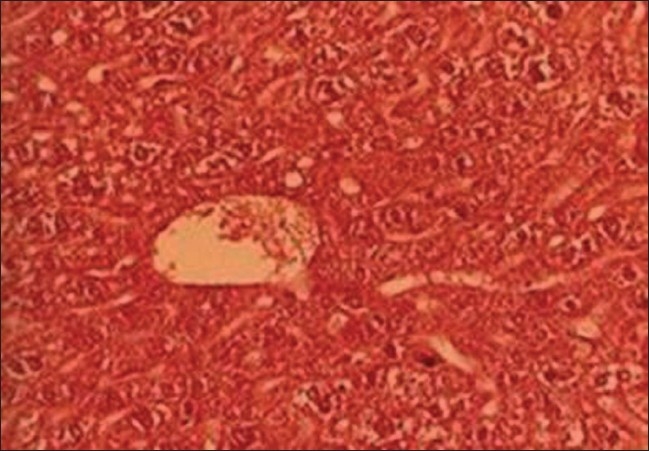
T.S. liver of TC (stem) treated group III (H and E ×66)

**Figure 4:**
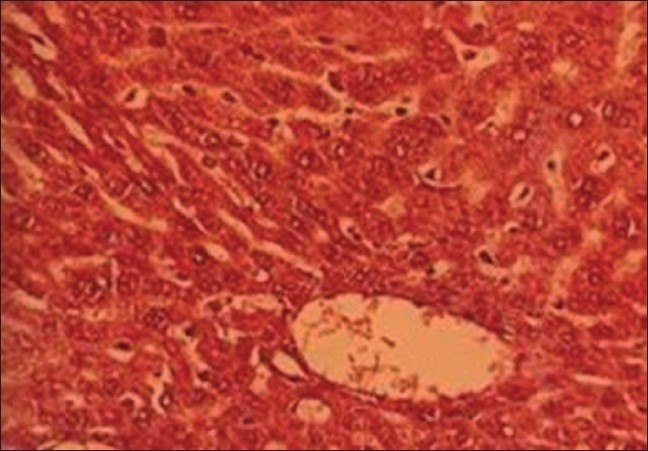
T.S. liver of TC (leaf) treated group IV (H and E ×66)

*Group III and IV (TC stem and leaf treated animals)*: Animals from these groups showed no histological differences when compared with group I, except low vacuolation and disorganization, which both TC extracts produced protective effects in this organ against lead toxicity [Figures 3 and 4].

**Figure 5:**
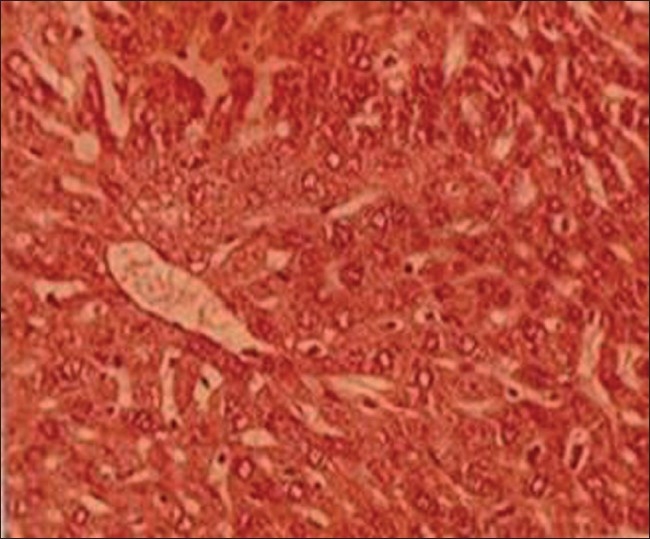
T.S. liver of TC (stem)+lead-treated group V (H and E ×66)

**Figure 6:**
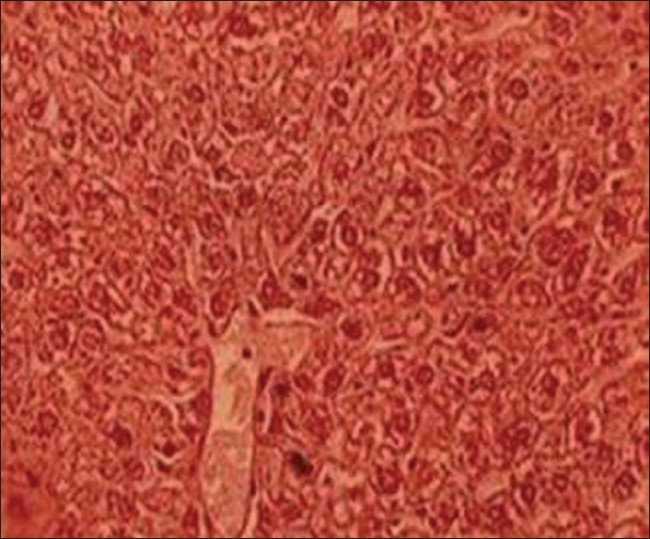
T.S. liver of TC (leaf)+lead-treated group VI (H and E ×66)

*Group V and VI (TC stem+ lead and TC leaf+ lead treated animals)*: When groups V and VI were compared with group II (lead treated), it was noticed that TC extracts retained hepatic architecture and was able to diminish the fibrosis, congestion, incidence of inflammatory cells infiltration centrilobular hepatocyte swelling, hepatocyte vacuolation, fatty changes, and hemorrhagic clots [Figure 5 and 6].

## DISCUSSION

### Effects on some enzymes of hepatic tissue

The changes in some hepatic biochemical enzymes following lead exposure either alone or in combination with *T. cordifolia* [Table 1] indicated a significant alteration in the peroxidative process. Lead is known to produce oxidative damage in the liver by enhancing peroxidation of lipid membranes[17] and lipid peroxidation is deleterious process solely carried out by free radicals.[23] In fact, LPO is an outcome of chain of events involving initiations, propagations, and termination reactions.[24] Unchecked peroxide decomposition of membrane lipid is catastrophic for living organism. The lipid peroxide produced is degraded to variety of products, including alkanols, hydroxyl alkanols, ketones, alkenes.[23] All these products inactivate cell constituents by oxidation or cause oxidation injury or damage by undergoing radical chain reaction ultimately leading to loss of membrane integrity.[17,25–28] Usually the deleterious effects of oxidative stress are counteracted by endogenous antioxidant enzymes mainly superoxide dismutase (SOD) and CAT.[29,30]

**Table 1:** Protective effects of *Tinospora cordifolia* on some hepatic biochemical parameters in lead-exposed mice

Groups	SOD	CAT	AST	ALT	ALP	ACP	Hepatic lead
Control (untreated animals)	6.25±0.14	161±0.84	2.28±0.04	3.14±0.06	15.1±0.24	1.73±0.08	0.07±0.07
Pb	2.27±0.16^a^	117±2.22^a^	5.67±0.19^a^	7.80±0.15^a^	18.1±0.54^a^	5.61±0.07^a^	2.14±0.17^a^
TC (stem)	6.84±0.33	183±2.22^a^	3.62±0.11	3.21±0.08	15.3±0.12^a^	3.14±0.06	0.17±0.03
TC (leaf)	5.61±0.07	148±3.35	3.72±0.22	3.62±0.11	15.9±0.68	2.52±0.04	0.21±0.01
TC (stem)+Pb	4.83±0.06^b^	161±4.99^b^	3.11±0.59^b^	4.67±0.05^b^	13.2±0.33^b^	2.46±0.01^b^	1.14±0.18^b^
TC (leaf)+ Pb	3.62±0.13	153±1.34^b^	3.21±0.08^b^	4.04±0.22^b^	11.7±0.26^b^	2.23±0.06^b^	1.34±0.09^b^

TC, *Tinospora cordifolia*; Pb, lead; SOD, superoxide dismutase (units/min/mg protein); CAT, catalase (μm H_2_O_2_ consumed/min/mg protein); AST, aspartate aminotransferase (per min per mg protein); ALT, alanine aminotransferase (per min per mg protein); ALP, alkaline phosphatase (one king Armstrong unit 1 UI-1); ACP, acid phosphatase (one king Armstrong unit/ 1 UI-1); lead liver concentration (μg/g); Values are mean±S.E, *n* = 6;

a *p*< 0.05 compared to control (untreated) animals;

b *p*< 0.05 compared to lead-exposed animals

In the present study, lead exposure produced significant adverse effect on the status of liver, which is evidenced by significant alterations of some tissue enzymes levels. Levels of SOD and CAT enzymes were reduced by lead, thus rendering the tissue to the peroxidative damage. Marked elevation of AST, ALT, ALP, and ACP in liver tissue indicates damage to the tissue. Administration of *T. cordifolia* extract to some extent could reduce oxidative stress/injury indicating its protective role in liver tissue. This was supported by slight increase or decrease in the activities of some liver enzymes by the drug. Reports have also suggested that *T. cordifolia* exhibit antioxidant[31,32] and hepatoprotective activities.[33] Interestingly, when the *T. cordifolia* extract were administered along with the lead, almost all liver enzymes to some extent improved as compared to lead-treated animals, supporting the antioxidant and hepatoprotective role of plant extracts. Earlier studies with known antioxidant such as vitamin E also showed that it helps in counteracting lead-induced oxidative damage.[17] Alpha tocopherol (vitamin E) and ascorbic (vitamin C) are known to decrease free radical generation by directly quenching the lipidperoxyl radicals and by increasing the SOD and CAT activities.[16,17,34] Pandey and Flora[35] reported that lipoic acid in combination with Succimer thiol chelators reduces lead-induced oxidative stress. Some studies also show that many herbs help in reducing lead-induced oxidative damage.[28,36,37]

### Effects on histology of hepatic tissue

Lead exposure produced pronounced hepatic histopathology evidenced by histological alternations in liver including focal necrosis with inflammatory cells, congestion at places, sinusoids not patent, centrilobular swelling, hepatocyte vacuolation and swelling, parenchyma disorganization, dilation of the inter hepatocyte space, and hemorrhagic clots. To some extent both TC extracts produced protective effects in this organ against lead toxicity. When TC extracts with lead administered, it retained hepatic architecture and was able to diminish the fibrosis, congestion, incidence of inflammatory cells infiltration of centrilobular hepatocyte swelling, hepatocyte vacuolation, fatty changes, and hemorrhagic clots. These findings are in support with Bishayi *et al*.,[33] who revealed that *T. cordifolia* affords protection against xenobiotics-induced liver damage including fibrosis and stimulates liver regeneration.

From the present study it is evident that *T. cordifolia* is capable of scavenging lead-induced free radical generation. It thus appears that the drug may prove useful in treating/preventing lead toxicity to some extent.
